# Comprehensive evaluation of AlphaMissense predictions by evidence quantification for variants of uncertain significance

**DOI:** 10.3389/fgene.2024.1487608

**Published:** 2024-12-10

**Authors:** Amina Kurtovic-Kozaric, Lejla Delalic, Belma Mutapcic, Lejla Comor, Eric Siciliano, Mark J. Kiel

**Affiliations:** Genomenon, Ann Arbor, MI, United States

**Keywords:** variants of unknown significance, VUS, AlphaMissense, ACMG/AMP classification, variant classification

## Abstract

Accurate variant classification is critical for genetic diagnosis. Variants without clear classification, known as “variants of uncertain significance” (VUS), pose a significant diagnostic challenge. This study examines AlphaMissense performance in variant classification, specifically for VUS. A systematic comparison between AlphaMissense predictions and predictions based on curated evidence according to the ACMG/AMP classification guidelines was conducted for 5845 missense variants in 59 genes associated with representative Mendelian disorders. A framework for quantifying and modeling VUS pathogenicity was used to facilitate comparison. Manual reviewing classified 5845 variants as 4085 VUS, 1576 pathogenic/likely pathogenic, and 184 benign/likely benign. Pathogenicity predictions based on AlphaMissense and ACMG guidelines were concordant for 1887 variants (1352 pathogenic, 132 benign, and 403 VUS/ambiguous). The sensitivity and specificity of AlphaMissense predictions for pathogenicity were 92% and 78%. Moreover, the quantification of VUS evidence and heatmaps weakly correlated with the AlphaMissense score. For VUS without computational evidence, incorporating AlphaMissense changed the VUS quantification for 878 variants, while 56 were reclassified as likely pathogenic. When AlphaMissense replaced existing computational evidence for all VUS, 1709 variants changed quantified criteria while 63 were reclassified as likely pathogenic. Our research suggests that the augmentation of AlphaMissense with empirical evidence may improve performance by incorporating a quantitative framework to aid in VUS classification.

## Background

Advances in genomic technologies, particularly next-generation sequencing, have revolutionized the detection and analysis of germline variants, offering unprecedented precision in the exploration of the intricacies of the human genome and its relationship to disease. Initiatives such as the Human Genome Project, 1000 Genomes Project, UK Biobank, gnomAD, and All of Us have significantly contributed to cataloging genetic variations across diverse populations, providing valuable insights into the implications of germline variants for health (Lander et al., 2001; 1000 Genomes Project Consortium, 2015; Koenig et al., 2024).

In the past, the identification of inherited disorders relied on direct evidence such as phenotype observation, the presence of the variant alongside the disease, or personal and family medical history. The emergence of gene sequencing and the rapid increase in variant data made it imperative to establish guidelines for variant classification. The American College of Medical Genetics (ACMG) was among the pioneers in offering recommendations and by introducing five categories of interpretation based on variant reporting and its association with Mendelian diseases (Slavin et al., 2019). The ACMG guidelines categorize variants into five tiers based on their pathogenicity, ranging from the least to most severe: benign, likely benign, variants of uncertain significance (VUS), likely pathogenic, and pathogenic. Clinically actionable variants, with implications for counseling and clinical care, fall into the pathogenic and likely pathogenic categories. On the other hand, variants in the benign, likely benign, and VUS categories are considered clinically non-actionable. Variants may undergo reclassification as new information emerges about their phenotypic effects. Such reclassification can significantly impact patient care (Slavin et al., 2019).

The accurate and standardized classification of genetic variants is vital to advancing both clinical practice and scientific research, contributing to enhanced patient care and a deeper understanding of the genetic underpinnings of diseases. Although many genetic variants possess known clinical significance, a substantial number fall into the VUS category, presenting formidable challenges to clinicians, geneticists, and patients due to the ambiguity surrounding their impact on health and disease. VUS encompass genetic variations identified through sequencing that lack sufficient evidence for definitive classification as either pathogenic or benign. Even when present in relevant genes that match the clinical circumstances of the patient, the presence of a VUS does not confirm a genetic diagnosis, highlighting the complexities in interpreting these variants (Joynt et al., 2021). Challenges arise from insufficient evidence, limited functional data, and divergent evaluations. The complex nature of VUS, often in non-coding regions or present as missense variants, hinders functional assessment and limits predictive power. Divergent approaches between scientists and clinicians, coupled with inconsistent reporting, contribute to classification difficulties. Enhancing VUS classification involves genetic testing on family members, *in vitro* tests, and using clinical databases and predictive algorithms (Federici and Soddu, 2020).

VUS often undergo category reclassification, particularly in underrepresented minority populations or with the promulgation of new clinical findings or functional studies, occurring months to years after the initial classification. Studies indicate frequent downgrades during reclassification, thus emphasizing the evolving understanding of these variants. However, VUS may be upgraded to pathogenic classification after additional functional or clinical evidence is gathered. A VUS outcome derived from clinical genetic testing should not be used for immediate management decisions but should be considered an indication of the absence of a pathogenic mutation at that moment (Slavin et al., 2019; Hoffman-Andrews, 2018).

In 2020, the NHGRI predicted the elimination of “variants of uncertain significance (VUS)” by making genomic variant predictions more predictable. However, as of 1 August 2023, the composition of the ClinVar database demonstrates the scale of the challenge comprising 36% VUS and 5% conflicting classifications among over 2 million variants. This VUS burden is mirrored in genetic testing reports, with 32% of inconclusive results lacking clear explanations for associated diseases. Despite these challenges, there remains a belief in the potential of largely eliminating VUS, emphasizing the importance of present decisions for achieving this goal by 2030. They argue that investing in eliminating VUS is crucial to advancing precision genomic medicine (Fowler and Rehm, 2024).

The ongoing challenge in human genetics lies in classifying variants, especially missense variants, where only a small fraction have been clinically categorized. Machine learning approaches offer a potential solution by leveraging biological data patterns, notably from evolutionary conservation in genetic residues and/or 3D conformation and structural protein modeling, to predict the pathogenicity of unannotated variants, thus addressing the gap in variant interpretation (Cheng et al., 2023). ACMG/AMP guidelines utilize this *in silico* evidence, such as pathogenic (PP3) and benign (BP4) evidence, for variant classification, but they only provide a small, supporting contribution to overall pathogenicity determination. However, the impact of these criteria on variant classification outcomes remains inadequately studied, particularly for missense, splice site, or non-coding variants, where the choice of *in silico* predictors becomes crucial. The challenge intensifies as concordance among predictors becomes more difficult to achieve with the inclusion of multiple tools, highlighting the need for comprehensive and standardized approaches in variant assessment (Burke et al., 2022; Barboso et al., 1987; Wilcox et al., 2022).

Recent research indicates that *in silico* tools, particularly pathogenicity classifiers, could enhance the accuracy of variant classification, effectively shortening the time to definitive diagnosing patients with these VUS variants without compromising diagnostic accuracy. These tools use artificial intelligence (AI) algorithms to quickly analyze variant descriptors and classify them as pathogenic or benign (Porras et al., 2024). Among these, AlphaMissense has emerged as a cutting-edge tool, utilizing AI methods trained on predicted protein structures. AlphaMissense leverages deep learning techniques derived from AlphaFold2, focusing on predicting the pathogenicity of missense variants in human and primate populations. Despite not being explicitly trained on genetic and experimental data, the model outperforms existing methods, assigning pathogenicity scores to genes that correlate with their cell essentiality. The comprehensive database provided by AlphaMissense categorizes 89% of missense variants as likely benign or likely pathogenic, demonstrating its robustness in predicting variant effects (Cheng et al., 2023).

Whereas AlphaMissense, with its innovative approach combining AI, structural information, and evolutionary conservation, has shown significant promise in predicting the functional impact of missense variants, the validity of these predictions has not been adequately assessed. To test AlphaMissense’s innovative approach in practice, we selected 59 genes that had been comprehensively curated by the Mastermind genomic evidence platform, providing a large number of testable missense variants. The primary objective of this study was to evaluate the predictive accuracy and reliability of AlphaMissense in characterizing missense variants in a curated set of 59 representative genes associated with neurological, musculoskeletal, and/or neuromuscular disorders through a comprehensive comparative analysis between AlphaMissense predictions and the application of clinical-grade, gold-standard manual interpretations of evidence according to the ACMG classification as enhanced by Mastermind.

## Methods

### Genes and variant selection

We undertook a comparative analysis between Google DeepMind’s AlphaMissense predictions and the ACMG classification (Richards et al., 2015) adapted by Genomenon through its genomic intelligence platform—Mastermind (MM). Our study focused on missense variants from a curated set of 59 genes comprehensively curated in Mastermind to increase the yield of testable missense variants: *AGRN, BPNT2, MUSK, MYO9A, SCN4A, SNAP25, ABHD16A, ACSL4, ANKLE2, AP3B2, ASAH1, BEAN1, BRWD3, CABP4, CC2D1A, CDC42BPB, CEP135, CRADD, DDHD1, FAM126A, FOXP1, GRIN2A, KDM5C, NBEA, NF1, PLA2G6, PRRT2, SACS, SCN8A, SLC52A3, SNIP1, STAG2, TAF2, WDR45, COQ8A, GLRB, SLC6A5, ALG14, ASCC1, CAPN3, CHAT, CHRNB1, CHRND, CHRNE, COL13A1, COL6A3, COLQ, DOK7, DPM3, DYSF, FKRP, GCH1, GFPT1, LRP4, PREPL, RYR1, SLC18A3, SLC5A7,* and *VAMP1*. Variants in these genes are associated with neurological, musculoskeletal, and/or neuromuscular disorders.

### AlphaMissense data extraction

AlphaMissense (AM) predictions of missense variants in selected genes were extracted from Cheng et al. (2023) including the AM score and pathogenicity classification (pathogenic, benign, and ambiguous). AM range was defined as: 0–0.33, benign; 0.34–0.564, ambiguous; 0.565–1, pathogenic (Cheng et al., 2023). ACMG classification included pathogenic/likely pathogenic, benign/likely benign, and VUS classifications (Richards et al., 2015). When comparing AlphaMissense to adapted ACMG classification conducted by expert review in Mastermind (Genomenon), AlphaMissense pathogenic prediction was taken as equivalent to pathogenic/likely pathogenic by ACMG classification, benign by AlphaMissense was taken as equivalent to benign/likely benign by ACMG, and AlphaMissense ambiguous prediction was taken as equivalent to VUS by ACMG.

### ACMG classification

The curation process involved a team of expert curators who manually curated selected genes according to the standards set by the American College of Medical Genetics and Association of Molecular Pathologists (ACMG/AMP) into benign, likely benign, variant of unknown significance (VUS), likely pathogenic, and pathogenic classifications (Richards et al., 2015). This interpretation process considered population frequencies derived from gnomAD v2.1.1, clinical and functional studies from literature, computational predictions of the effect of missense variants derived from REVEL, PolyPhen-2, MutationTaster2, and SIFT, and computational predictions of splicing defects for single nucleotide variants derived from dbscSNV.

### Comparison of AlphaMissense predictions to ACMG classification

Sensitivity, specificity, positive predictive values (PPVs), and negative predictive values (NPVs) were calculated for all genes and individual genes (*RYR1, FKRP, DOK7, NF1, GRIN2A, SCN4A*) (Trevethan (2017). Sensitivity was calculated as [*a*/(*a*+*c*)]×100, specificity was calculated as [*d*/(*b* + *d*)]×100, PPV was calculated as [*a*/(*a*+*b*)]×100, and NPV was calculated as [*d*/(*c* + *d*)]×100, where *a* represents variants predicted as pathogenic in AlphaMissense and by ACMG guidelines, *b* represents variants predicted as pathogenic by AlphaMissense but not by ACMG, *c* represents variants that are not pathogenic by AlphaMissense but pathogenic by ACMG, and *d* represents those variants that are not pathogenic (benign) by AlphaMissense and by ACMG guidelines. For most genes, sensitivity, specificity, PPV, and NPV were not calculated on the individual level due to the low number of missense variants which was insufficient to generate representable data. True positive (TP) values represent variants predicted as pathogenic in AM and classified as pathogenic/likely pathogenic by ACMG classification. False positive (FP) values were defined as variants predicted pathogenic in AlphaMissense but classified as benign by ACMG. Variants that were predicted as benign in AM but reached pathogenic/likely pathogenic classification by ACMG were defined as false negative (FN), while true negative (TN) values represent variants identified as benign by both AlphaMissense and ACMG assessments. Additionally, sensitivity, specificity, PPV and NPV were calculated for all genes by comparing pathogenic and not pathogenic (VUS) variants (in this case, TP and TN values were variants classified pathogenic/likely pathogenic or benign/likely benign by both AlphaMissense and ACMG, respectively; FN—variants called ambiguous by AlphaMissense but pathogenic/likely pathogenic by ACMG, and *vice versa* for FP). AlphaMissense (AM) and Mastermind (MM) were compared by creating different comparison groups of Mastermind and AlphaMissense data as follows: variants designated as pathogenic or benign both by AM and MM (PATH-path and BEN-ben, respectively), variants classified as pathogenic by MM but benign by AM, and *vice versa* (PATH-ben and BEN-path, respectively), variants classified as VUS by MM and ambiguous (VUS-amb), pathogenic (VUS-path) or benign by AM (VUS-ben), and finally data concerning variants designated as pathogenic or benign by MM and ambiguous by AM (PATH-amb and BEN-amb, respectively).

### Quantification of ACMG classification using a points-based system

We collected and analyzed individual ACMG evidence and evidence groups assigned to each variant (n = 5,845). The evidence groups differed by strength—benign standalone (BA), benign strong (BS), benign supporting (BP), pathogenic very strong (PVS), pathogenic strong (PS), pathogenic moderate (PM), and pathogenic supporting (PP) evidence—and type—population, computational, functional, clinical, and molecular impact.

To quantify changes in variant classification, we used a point system based on the Bayesian framework described in Tavtigian et al. (2020). Each evidence category by strength (BA, BS, BP, PVS, PS, PM, PP) was assigned a point value: −4 points were assigned to BA and BS, −1 points to BP, 0 points to indeterminate, 1 point to PP, 2 points to PM, 4 points to PS, and 8 points to PVS evidence groups.

We first focused on VUS variants (n = 1,141) classified by ACMG in Mastermind without any assigned computational evidence. We utilized AlphaMissense predictions as computational evidence, supporting either benign or pathogenic (BP4 or PP3). The aim was to investigate the influence of incorporating AlphaMissense predictions into the variant interpretation process on the final classifications of these variants. To explore how AlphaMissense could impact the change in the final classification of all VUS, we replaced the existing computational evidence with AlphaMissense predictions for the remaining 2944 VUS variants classified by ACMG criteria in Mastermind. Similar analysis was also conducted for all variants, where all computational evidence was removed and replaced with AlphaMissense predictions.

### Statistical analysis

Statistical analysis (chi square test and confidence intervals), box plot, density distribution bell curve, stacked graphs, and a scatter graph were generated using R studio. Heat maps were generated in Excel and Adobe InDesign.

## Results

### AlphaMissense variant predictions compared to ACMG classification

**FIGURE 1:**
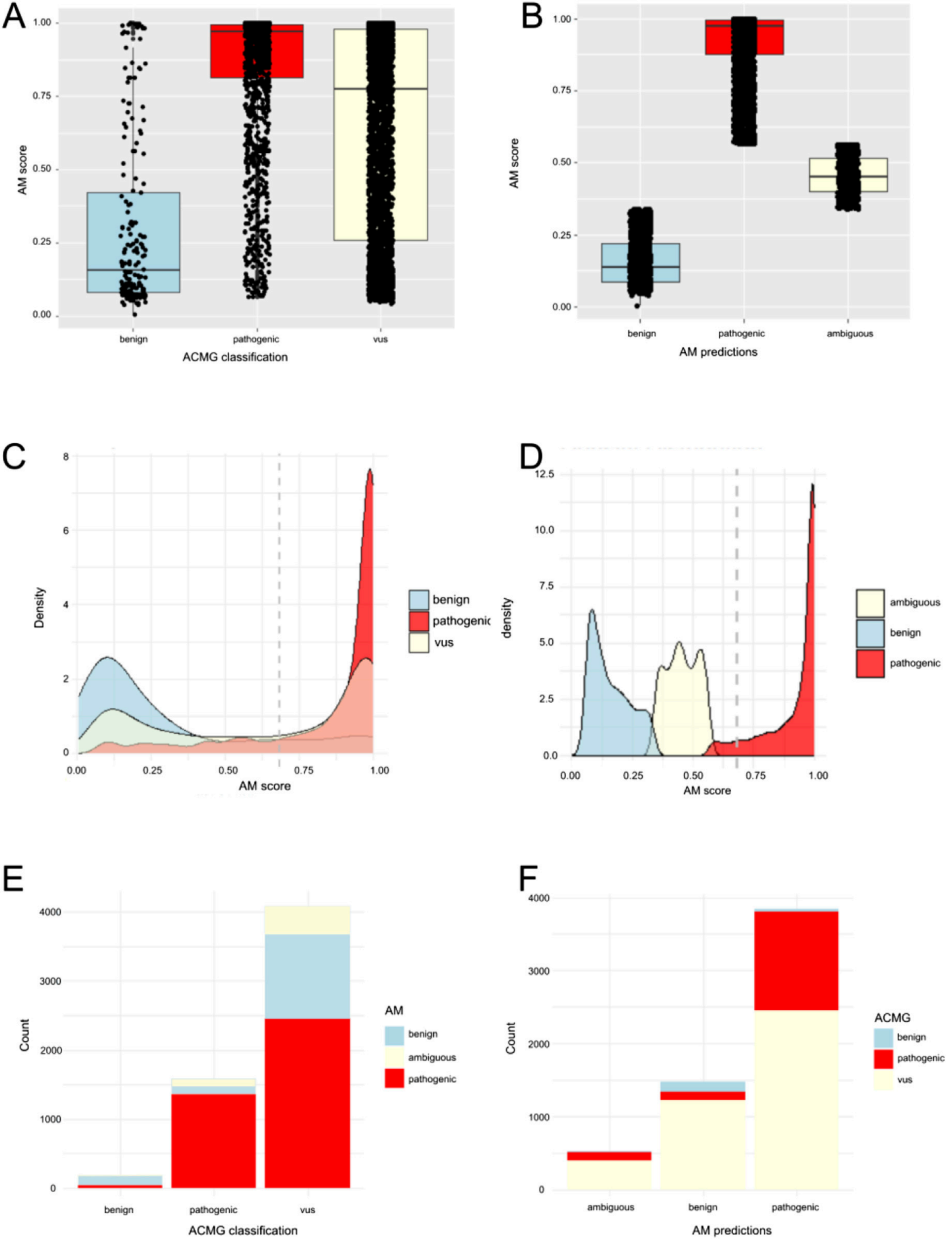
Comparison of AlphaMissense (AM) predictions to Mastermind (MM) classifications. **(A)** Distribution of AM scores among variants classified as pathogenic (including likely pathogenic n = 1,576), benign (including likely benign n = 184), or VUS (n = 4,085) presented as box-plot. **(B)** Distribution of AM scores among AM predictions for pathogenic (n = 3,848), benign (n = 1,472), and ambiguous variants (n = 524). **(C)** Density distribution bell curve of AM score among variants classified as pathogenic (including likely pathogenic n = 1,576), benign (including likely benign n = 184), or VUS (n = 4,085). **(D)** Density distribution bell curve of AM score among AM predictions for pathogenic (n = 3,848), benign (n = 1,472), and ambiguous variants (n = 524). **(E)** Ratio of MM classified variants, predicted as benign, pathogenic, or ambiguous by AM. **(F)**. Ratio of AM predicted variants classified as benign (including likely benign), VUS, or pathogenic (including likely pathogenic) by MM.

We selected 59 genes associated with musculoskeletal, neuromuscular, and/or neurological disorders as a representative dataset spanning a variety of different disease mechanisms and inheritances patterns (Table 1). Isolating missense variants revealed a total of 5845 variants, with 1576 classified as pathogenic/likely pathogenic, 184 as benign/likely benign, and 4,085 as VUS by ACMG classification in Mastermind (Supplementary Table S1). Thus, 70% of variants were VUS variants in our cohort. To compare the standard ACMG classification to AlphaMissense predictions (pathogenic, ambiguous, benign), we shortened the five-level ACMG classification to three levels for easier comparison (the pathogenic/likely pathogenic were classified as “pathogenic” and benign/likely benign were classified as “benign”). When AlphaMissense predictions were extracted for all missense variants, 3848 variants (66%) were classified as pathogenic, 1472 (25%) were benign, and 525 (9%) were ambiguous. The comparison of AlphaMissense predictions to ACMG classification is shown in Figure 1. The mean AlphaMissense score for benign variants was 0.3 (CI: 0.26–0.35), 0.85 (CI 0.84–0.86) for pathogenic and 0.64 (CI 0.63–0.65) for VUS variants. Benign and pathogenic classification by ACMG correlated well with AlphaMissense scores (*p* < 0.01); higher AlphaMissense scores were associated with pathogenic variants by ACMG classification, and lower scores were associated with benign variants (*p* < 0.01; Figures 1A, B). Using a density plot, VUS variants showed a bimodal (saddle) density distribution of AlphaMissense scores, with the majority of variants having either lower or higher scores (Figures 1C, D).

**TABLE 1:** Diseases, associated genes, and inheritance patterns evaluated in this study (n = 60).

Disease	Gene	Category	Inheritance pattern
Congenital myasthenic syndrome	*AGRN*	Musculoskeletal disease	AR
Skeletal dysplasia	*BPNT2*	Musculoskeletal disease	AR
Myasthenic syndrome	*MUSK*	Musculoskeletal disease	AR
Congenital myasthenic syndrome	*MYO9A*	Musculoskeletal disease	AR
Paramyotonia congenita	*SCN4A*	Musculoskeletal disease	AD; AR
Congenital myasthenic syndrome	*SNAP25*	Musculoskeletal disease	AD
Hereditary spastic paraplegia	*ABHD16A*	Neurological	AR
Intellectual disability	*ACSL4*	Neurological	XL
Microcephaly	*ANKLE2*	Neurological	AR
AP3B2-related epilepsy and neurodevelopmental disorders	*AP3B2*	Neurological	AR
ASAH1-related disorders	*ASAH1*	Neurological	AR
Spinocerebellar ataxia	*BEAN1*	Neurological	AD
Intellectual developmental disorder	*BRWD3*	Neurological	XL
Cone-rod dystrophy; epilepsy	*CABP4*	Neurological	AR
Neurodevelopmental disorder; heterotaxy	*CC2D1A*	Neurological	AR
CDC42BPB-related neurodevelopmental disorder	*CDC42BPB*	Neurological	AD
Microcephaly	*CEP135*	Neurological	AR
Intellectual disability	*CRADD*	Neurological	AR
Hereditary spastic paraplegia	*DDHD1*	Neurological	AR
Hypomyelinating leukodystrophy	*FAM126A*	Neurological	AR
FOXP1-related syndrome	*FOXP1*	Neurological	AD
Landau–Kleffner syndrome	*GRIN2A*	Neurological	AD
Intellectual developmental disorder, Claes–Jensen type	*KDM5C*	Neurological	XL
Neurodevelopmental disorder with or without early-onset generalized epilepsy	*NBEA*	Neurological	AD
Neurofibromatosis type 1 (NF1)	*NF1*	Neurological	AD
Spinocerebellar ataxia	*NOP56*	Neurological	AD
Infantile neuroaxonal dystrophy	*PLA2G6*	Neurological	AR
PRRT2-associated paroxysmal movement disorders	*PRRT2*	Neurological	AD
Autosomal recessive spastic ataxia of Charlevoix–Saguenay	*SACS*	Neurological	AR
SCN8A-related epilepsy and neurodevelopmental disorders	*SCN8A*	Neurological	AD
Riboflavin transporter deficiency	*SLC52A3*	Neurological	AR
Neurodevelopmental disorder with hypotonia, craniofacial abnormalities, and seizures (NEDHCS)	*SNIP1*	Neurological	AR
STAG2-related neurodevelopmental disorders	*STAG2*	Neurological	XL
Intellectual disability	*TAF2*	Neurological	AR
Beta-propeller protein-associated neurodegeneration (BPAN)	*WDR45*	Neurological	XL
Coenzyme Q10 deficiency	*COQ8A*	Neurological	AR
Hereditary hyperekplexia	*GLRB*	Neurological	AR
Hyperekplexia	*SLC6A5*	Neurological	AD; AR
Congenital myasthenic syndrome	*ALG14*	Neuromuscular	AR
Spinal muscular atrophy; esophageal cancer	*ASCC1*	Neuromuscular	AR
Limb-girdle disease	*CAPN3*	Neuromuscular	AD; AR
Congenital myasthenic syndrome	*CHAT*	Neuromuscular	AD, AR
Congenital myasthenic syndrome	*CHRNB1*	Neuromuscular	AD
Congenital myasthenic syndrome	*CHRND*	Neuromuscular	AD; AR
Congenital myasthenic syndrome	*CHRNE*	Neuromuscular	AD; AR
Congenital myasthenic syndrome	*COL13A1*	Neuromuscular	AR
COL6A3-related myopathy and dystonia disorders	*COL6A3*	Neuromuscular	AD: AR
Congenital myasthenic syndrome	*COLQ*	Neuromuscular	AD, AR
Congenital myasthenic syndrome	*DOK7*	Neuromuscular	AD, AR
DPM3-related muscular dystrophy	*DPM3*	Neuromuscular	AR
Dysferlinopathy	*DYSF*	Neuromuscular	AR
Muscular dystrophies	*FKRP*	Neuromuscular	AR
Dopa-responsive dystonia with or without hyperphenylalaninemia	*GCH1*	Neuromuscular	AD: AR
Congenital myasthenic syndrome	*GFPT1*	Neuromuscular	AD, AR
Cenani–Lenz syndactyly syndrome; sclerosteosis; congenital myasthenic syndrome	*LRP4*	Neuromuscular	AD; AR
Congenital myasthenic syndrome	*PREPL*	Neuromuscular	AR
RYR1-related myopathy disorders; malignant hyperthermia	*RYR1*	Neuromuscular	AD
Congenital myasthenic syndrome	*SLC18A3*	Neuromuscular	AR
Congenital myasthenic syndrome; distal hereditary motor neuropathy	*SLC5A7*	Neuromuscular	AD; AR
Congenital myasthenic syndrome	*VAMP1*	Neuromuscular	AD; AR

Out of 5845 variants, AlphaMissense prediction and ACMG classification were concordant in 1887 variants (1352 variants were pathogenic in both, 132 were benign in both, and 403 were VUS/ambiguous for both). Regarding discrepancies, when we analyzed pathogenic ACMG-classified variants, a minority of pathogenic variants (224 variants out of 1576) showed discrepant classification (108 were ambiguous and 116 were benign in AlphaMissense) (Figures 1E, F; Table 2). When we analyzed benign ACMG-classified variants, most were also benign in AlphaMissense (132 variants out of 184 benign variants), 38 benign variants were predicted as pathogenic, and 14 were predicted as ambiguous by AlphaMissense. When we analyzed VUS ACMG-classified variants (n = 4085), which were the largest number of variants in our cohort, 2458 VUS variants were classified as pathogenic and 1224 as benign by AlphaMissense, revealing that a majority of VUS variants carry pathogenic prediction in AlphaMissense.

**TABLE 2:** Comparison between AlphaMissense predictions and ACMG classification by Mastermind. (A) Number of pathogenic, VUS, and benign variants classified by ACMG criteria compared to their AlphaMissense predictions. True positive (TP) values represent variants predicted as pathogenic in AM and classified as pathogenic/likely pathogenic by Mastermind. False positive (FP) values defined as variants predicted pathogenic in AlphaMissense but classified as benign by Mastermind. Variants predicted as benign in AM but reached pathogenic/likely pathogenic classification by MM were defined as FN, while TN values represent variants identified as benign by both AM and MM assessments. (B) Sensitivity, specificity, positive predictive values (PPVs), and negative predictive values (NPVs) were calculated for all genes by comparing pathogenic and not pathogenic (benign or VUS) variants.

A			ACMG Mastermind classification						
			Pathogenic		VUS		Benign		
AlphaMissense prediction	Pathogenic		1,352		2,458		38		
	Ambiguous		108		403		14		
	Benign		116		1,224		132		

B	Sensitivity		Specificity		PPV		NPV		
ACMG Path/Ben versus AlphaMissense Path/Ben	92%		78%		97%		53%		
ACMG Pathogenic/VUS versus AlphaMissense Path/Amb	93%		14%		36%		79%		
ACMG Pathogenic/non-pathogenic (VUS + benign) versus AlphaMissense pathogenic/non-pathogenic (ambiguous + benign)	86%		41%		35%		89%		

Sensitivity, specificity, positive predictive value (PPV), and negative predictive value (NPV) are shown in Table 2. Sensitivity for predicting pathogenic variants by AlphaMissense was 92% and specificity was 78% (*p* < 0.05). The overall PPV was 97%, while NPV reached 53%. When comparing ACMG pathogenic and all non-pathogenic variants (i.e., VUS and benign) to AlphaMissense pathogenic and non-pathogenic variants (i.e., ambiguous and benign), sensitivity was 86%, but specificity was 41%, and PPV and NPV were 35% and 89%, respectively (Table 2). An individual evaluation of sensitivity, specificity, PPV, and NPV was performed for six genes characterized by a higher number of interpreted missense variants (Supplementary Table S2). Sensitivity and PPV were all above 90%, but specificity ranged from 33% to 69% and NPV from 25% to 90%.

AlphaMissense scores for 5845 variants were compared to REVEL (rare exome variant ensemble learner) scores, which is an ensemble method for predicting the pathogenicity of missense variants. AlphaMissense scores correlated highly with REVEL scores (Spearman rank correlation coefficient, R > 0.6).

### AlphaMissense predictions compared to the type of evidence criteria

**FIGURE 2:**
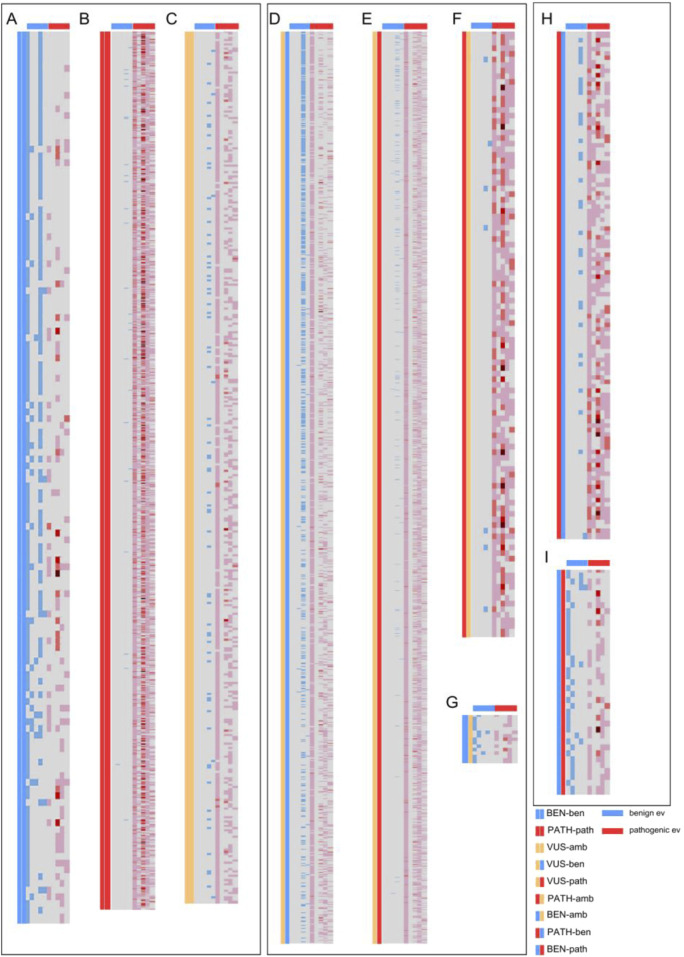
Heatmaps showcasing the individual ACMG evidence (grouped by type) assigned for each missense variant from the 59 evaluated genes. Each row represents a missense variant from one of the 59 genes. Columns represent, from left to right: two columns depicting the comparison groups, benign population, benign functional and allelic, benign clinical, benign computational, and benign molecular impact evidence, as marked by the blue horizontal lines above; and pathogenic population, pathogenic functional and allelic, pathogenic clinical, pathogenic computational, and pathogenic molecular impact evidence, as marked by the red horizontal lines. The blue and red colors in the columns represent assigned evidence, with darker red colors depicting multiple evidence assigned from that evidence group. **(A)** Comparison of benign variants by ACMG classification and AlphaMissense. **(B)** Comparison of pathogenic variants by ACMG classification and AlphaMissense. **(C)** Comparison of VUS/ambiguous variants by ACMG classification and AlphaMissense. **(D)** Comparison of VUS by ACMG and benign by AlphaMissense. **(E)** Comparison of VUS by ACMG and pathogenic by AlphaMissense. **(F)** Comparison of pathogenic by ACMG and VUS by AlphaMissense. **(G)** Comparison of benign by ACMG and VUS by AlphaMissense. **(H)** Comparison of pathogenic by ACMG and benign by AlphaMissense. **(I)** Comparison of benign by ACMG and pathogenic by AlphaMissense.

To further understand the value of AlphaMissense predictions in variant classification, we decided to segregate each ACMG call according to individual evidence categories. Individual ACMG evidence sorted by type (population, functional, computational, clinical, and molecular impact) assigned to each variant were evaluated in relation to both AlphaMissense prediction and ACMG classification (Figure 2). Each heatmap represents the comparison between ACMG classification (pathogenic, benign, or VUS) and AlphaMissense prediction (pathogenic, benign, or ambiguous), where each row is a unique variant. We compared concordant calls between ACMG classification and AlphaMissense predictions—benign ACMG classification versus benign AlphaMissense (BEN-ben, Figure 2A), pathogenic ACMG versus pathogenic AlphaMissense (PATH-path, Figure 2B), and VUS ACMG versus ambiguous AlphaMissense predictions (VUS-amb, Figure 2C). This revealed a clear association between evidence criteria and AlphaMissense calls. For VUS versus ambiguous, the evidence showed either a lack of evidence assigned to a variant or the presence of both benign and pathogenic criteria which designated these variants as true VUS. For discordant calls, we compared VUS ACMG classification versus benign AlphaMissense (VUS-ben, Figure 2D), VUS ACMG classification versus pathogenic AlphaMissense (VUS-path, Figure 2E), pathogenic ACMG classification versus ambiguous AlphaMissense predictions (PATH-amb, Figure 2F), and benign ACMG classification versus ambiguous AlphaMissense predictions (BEN-amb, Figure 2G). For VUS ACMG classification versus AlphaMissense benign/pathogenic prediction comparison (Figures 2D, E), there is a clear concordance between the benign computational calls (evidence from other prediction tools) and benign AlphaMissense predictions, but the existence of pathogenic evidence such as population, clinical or molecular impact evidence classified the variants as VUS. Thus, when comparing ACMG VUS to AlphaMissense pathogenic prediction, the majority of variants have a pathogenic population and computational criteria, which are insufficient to push the classification from VUS to likely pathogenic. A small subset of variants has computational predictions and some have functional data pointing to their benignity. However, there is a trend where most variants with pathogenic AlphaMissense predictions also have pathogenic criteria assigned. Evidence from the comparison between pathogenic ACMG classification and ambiguous AlphaMissense implies that most ambiguous calls have pathogenic evidence associated with them, whereas the BEN-amb comparison group has both benign and pathogenic evidence assigned, with benign population evidence being sufficient in calling a benign classification with certainty. The opposite calls were also compared—pathogenic ACMG classification versus benign AlphaMissense (Figure 2H) and benign ACMG classification versus pathogenic AlphaMissense predictions (Figure 2I). Comparing ACMG pathogenic to AlphaMissense benign (n = 1,576), the majority of variants have pathogenic evidence in most categories with a subset of benign *in silico* predictions, suggesting that these AlphaMissense predictions may be false negatives. Comparing ACMG benign classification to AlphaMissense pathogenic predictions (n = 38), most variants have benign population criteria, making these AlphaMissense predictions likely false positives. Analyzing AlphaMissense ambiguous predictions (Figures 2C,F,G), the majority of variants have also VUS ACMG classification (n = 403), followed by pathogenic ACMG classification (n = 108); only a few variants have benign ACMG classification (n = 14), suggesting that ambiguous AlphaMissense predictions correlate well with VUS classification and tend to be associated with pathogenic ACMG classification.

### AlphaMissense predictions compared to the strength of evidence criteria

In addition to evidence type, individual ACMG calls were categorized by their strength (benign strong (BS), benign supporting (BP), pathogenic strong (PS), pathogenic moderate (PM), and pathogenic supporting (PP)). Each criterion was assigned to all evaluated missense variants that were differently classified or predicted by Mastermind and AlphaMissense (Tables 3, 4). The most commonly used evidence for VUS and pathogenic variants was PM (48%–54%), while for benign variants it was BS (37%–39%). If we analyze ACMG groups (VUS, pathogenic and benign), the distribution across evidence criteria was even for ACMG pathogenic and benign variants, except for VUS variants. For ACMG VUS variants, BP was used in 25% of variants with benign AlphaMissense prediction compared to 3% of VUS variants with pathogenic prediction, which is explained by the frequent use of computational BP4 evidence.

**TABLE 3:** Quantity of all individual ACMG evidence (grouped by type: population, functional and allelic, clinical, computational, and molecular data) assigned to all evaluated missense variants differently classified or predicted by Mastermind and AlphaMissense, respectively.

Mastermind classification	Alpha missense prediction	Number of variants	Population data		Functional and allelic data		Clinical data		Computational data		Molecular impact		Total
			Benign (BA1, BS1, and BS2)	Pathogenic (PS4, PM2, and PS4_M)	Benign (BS3 and BP2)	Pathogenic (PS3 and PM3)	Benign (BS4 and BP5)	Pathogenic (PP1, PP1_M, PP4, PS2, PM6, PPC, PPC HET, PPC HOM, and PPC COMHET)	Benign (BP4)	Pathogenic (PP3)	Benign (BP7, BP3, and BP1)	Pathogenic (PVS1, PS1, PM4, PM1, PM5, and PP2)	
VUS	Benign	1,224	0	1,101 (41%) 1,091 (1) 10 (2)	0	1 (0%) 1 (1)	5 (0%) 5 (1)	343 (13%) 284 (1) 54 (2) 4 (3) 1 (4)	579 (22%) 579 (1)	337 (13%) 337 (1)	11 (0%) 11 (1)	281 (11%) 263 (1) 18 (2)	2,658
	Pathogenic	2,458	0	2,435 (42%) 2,368 (1) 67 (2)	0	0	4 (0%) 4 (1)	899 (16%) 755 (1) 138 (2) 6 (3)	120 (2%) 120 (1)	1,643 (28%) 1,643 (1)	9 (0%) 9 (1)	657 (11%) 616 (1) 41 (2)	5,767
Pathogenic (PATH + LPATH)	Ambiguous	108	0	107 (27%) 76 (1) 31 (2)	0	45 (12%) 45 (1)	0	91 (23%) 36 (1) 43 (2) 6 (3) 6 (4)	6 (2%) 6 (1)	81 (21%) 81 (1)	1 (0%) 1 (1)	59 (15%) 48 (1) 11 (2)	390
	Benign	116	0	115 (28%) 88 (1) 27 (2)	0	49 (12%) 49 (1)	0	78 (19%) 40 (1) 22 (2) 13 (3) 3 (4)	30 ((7%) 30 (1)	67 (16%) 67 (1)	1 (0%) 1 (1)	68 (17%) 55 (1) 13 (2)	408
Benign (BEN + LBEN)	Pathogenic	38	23 (21%) 23 (1)	20 (18%) 20 (1)	13 (12%) 13 (1)	0	1 (1%) 1 (1)	19 (17%) 11 (1) 5 (2) 2 (3) 1 (4)	5 (5%) 5 (1)	22 (20%) 22 (1)	1 (1%) 1 (1)	6 (5%) 5 (1) 1 (2)	110
	Ambiguous	14	9 (23%) 9 (1)	6 (15%) 6 (1)	4 (10%) 4 (1)	0	1 (3%) 1 (1)	5 (13%) 3 (1) 2 (2)	1 (3%) 1 (1)	11 (28%) 11 (1)	0	2 (5%) 2 (1)	39
Evidence for all variants			144 (1)	5,545 5,130 (1) 414 (2) 1 (3)	36 (1)	640 639 (1) 1 (2)	16 (1)	2,717 1731 (1) 738 (2) 182 (3) 58 (4) 8 (5)	912 (1)	3,550 (1)	40 (1)	1845 1,652 (1) 193 (2)	

In Column 1, the Mastermind classification is presented, wherein variants classified as pathogenic and likely pathogenic fall under the Pathogenic category, and variants classified as benign and likely benign are categorized as Benign. Column 2 shows the AlphaMissense classification, and Column 3 indicates the total number of variants in each category. Columns 4–13 display ACMG evidence, according to Richards et al. (2015). The number of variants with one, two, three, or four items of evidence is shown in parenthesis.

**TABLE 4:** Quantity and percentage of all individual ACMG evidence items, categorized by their strength (benign strong (BS), benign supporting (BP), pathogenic strong (PS), pathogenic moderate (PM), and pathogenic supporting (PP)) assigned to all evaluated missense variants that were differently classified or predicted by Mastermind and AlphaMissense, respectively.

ACMG Classification	AlphaMissense classification	Number of variants	Evidence group by ACMG					Total
			BP	BS	PP	PM	PS	
VUS	Benign	1,224	595 (25%) 595 (1)	0	630 (27%) 480 (1) 134 (2) 16 (3)	1,132 (48%) 956 (1) 176 (2)	1 (0%) 1 (1)	2,358
	Pathogenic	2,458	133 (3%) 133 (1)	0	1952 (43%) 1,190 (1) 622 (2) 140 (3)	2,441 (54%) 1950 (1) 491 (2)	0	4,526
Pathogenic (PATH + LPATH)	Ambiguous	108	7 (3%) 7 (1)	0	97 (38%) 35 (1) 47 (2) 11 (3) 4 (4)	108 (42%) 28 (1) 52 (2) 26 (3) 2 (4)	44 (17%) 43 (1) 1 (2)	256
	Benign	116	31 (11%) 31 (1)	0	85 (30%) 40 (1) 37 (2) 6 (3) 2 (4)	115 (41%) 37 (1) 39 (2) 34 (3) 5 (4)	50 (18%) 50 (1)	281
Benign (BEN + LBEN)	Pathogenic	38	5 (5%) 4 (1) 1 (2)	34 (37%) 34 (1)	29 (31%) 21 (1) 7 (2) 1 (3)	24 (26%) 19 (1) 5 (2)	0	92
	Ambiguous	14	1 (3%) 1 (1)	12 (39%) 12 (1)	11 (35%) 7 (1) 3 (2) 1 (3)	7 (23%) 6 (1) 1 (2)	0	31
	Total per evidence group		772	46	2,804	3,827	95	

In Column 1, the Mastermind classification is presented, wherein variants classified as pathogenic and likely pathogenic fall under the Pathogenic category, and variants classified as benign and likely benign are categorized as Benign. Column 2 shows the Alphamissense classification, and Column 3 indicates the total number of variants in each category. Columns 4–8 display ACMG evidence, according to Richards et al. (2015), with Column 8 representing the total sum of all ACMG evidence per category. The number of variants with one, two, three, or four items of evidence is shown in parenthesis.

We set out to analyze and compare the already existing ACMG computational evidence in our cohort to AlphaMissense predictions. We found that out of 912 BP4-assigned benign variants in ACMG, 696 (76%) variants also had benign AlphaMissense prediction while 144 variants were pathogenic and 72 were ambiguous. Out of 3550 PP3-assigned variants, 2828 variants also had pathogenic AlphaMissense prediction (80%), while 430 variants had benign (12%) and 292 (8%) had ambiguous AlphaMissense prediction. If we analyze only VUS variants, 72% (n = 2,944) had computational evidence (764 VUS variants had BP4, while 2,180 of variants had PP3 evidence). A total of 1141 out of 4085 variants did not have an assigned computational call. Furthermore, among 764 VUS variants that had BP4-assigned evidence, 76% (n = 579) variants were also benign in AlphaMissense, while 120 variants were pathogenic and 65 were ambiguous. A total of 2180 VUS variants had PP3-assigned evidence and 75% (n = 1,643) also had pathogenic AlphaMissense prediction, while 337 were predicted benign and 200 variants were predicted ambiguous. In conclusion, 75% of VUS variants showed concordance between already used computational tools and AlphaMissense.

### Contribution of AlphaMissense predictions as computational evidence in variant interpretation and reclassification

**FIGURE 3:**
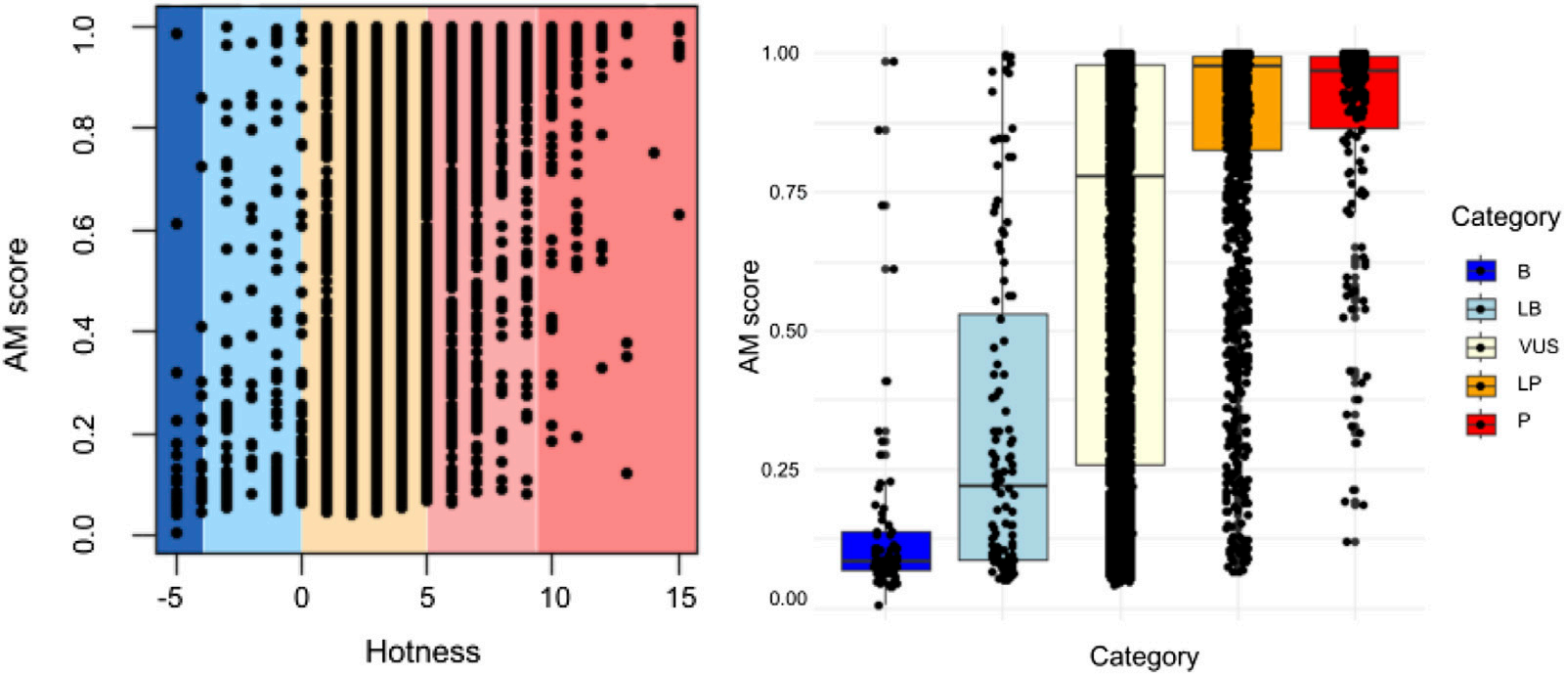
Quantification (points-based system) of variant pathogenicity using a Bayesian framework. Comparison of AlphaMissense scores for each classified variant is shown in relation to quantification (−8 to −4 as benign, −4 to −1 as likely benign, 0 to 5 as VUS, 6 to 9 as likely pathogenic, and ≥10 as pathogenic). All variants are presented, showcasing quantification by categories. A correlation exists between the AlphaMissense score and the point-based system, i.e., ACMG classification (*p* < 0.05).

Figure 3 depicts the quantification of variant pathogenicity using a points-based system within a Bayesian framework (Tavtigian et al., 2018). The quantification system uses a scale from ≤−4 to ≥10, where variants with ≤ −4 points designated as benign, −3 to −1 likely benign, 0–5 VUS, 6–9 likely pathogenic, and ≥10 as pathogenic. All variants (n = 5845) are presented, demonstrating quantification by categories and highlighting the correlation between the AlphaMissense score and the points-based system of ACMG classification. Quantified ACMG pathogenic and benign variants correlated well with high and low AlphaMissense scores, respectively.

We set out to analyze whether AlphaMissense may help in VUS reclassification of those variants that did not have computational evidence. Table 5 displays the impact of the addition of AlphaMissense predictions to computational evidence for VUS variants that *did not have* computational evidence. Evidence was quantified through a points-based system using a Bayesian framework (Tavtigian et al., 2018). It includes all 4085 variants with VUS classification, along with evidence groups from ACMG (20 groups, including various combinations of pathogenic very strong (PVS), pathogenic strong (PS), pathogenic moderate (PM), pathogenic supporting (PP), benign strong (BS), benign moderate (BP), and stand-alone support (BA) evidence).

**TABLE 5:** Impact of AlphaMissense predictions on VUS variants that did not have computational evidence. VUS variants were quantified through a points-based system using a Bayesian framework. The approach involves point adaptation (Tavtigian et al., 2020).

N of variants (n = 4,085)	PS	PM	PP	BP	BS	Quantification of ACMG classification	Quantification after addition of AlphaMissense benign prediction	Quantification after addition of AlphaMissense pathogenic prediction	Reclassified variants (n = 934)	Quantification changed	Quantification leading to reclassification
**24**	0	0	0	1	0	−1			0	No	No
**16**	0	0	0	0	0	0	−1 (n = 12)	1 (n = 1)	13	Yes	No
**19**	0	0	1	1	0	0			0	No	No
**35**	0	0	1	0	0	1	0 (n = 9)		9	Yes	No
**4**	0	0	2	1	0	1			0	No	No
**406**	0	1	0	1	0	1	0 (n = 4)	2 (n = 2)	6	Yes	No
**23**	0	0	2	0	0	2	1 (n = 1)		1	Yes	No
**546**	0	1	0	0	0	2	1 (n = 150)	3 (n = 318)	468	Yes	No
**203**	0	1	1	1	0	2			0	No	No
**1,111**	0	1	1	0	0	3		4 (n = 191)	191	Yes	No
**4**	0	0	3	0	0	3			0	No	No
**29**	0	1	2	1	0	3			0	No	No
**71**	0	2	0	1	0	3	2 (n = 1)	4 (n = 2)	3	Yes	No
**775**	0	1	2	0	0	4	3 (n = 6)	5 (n = 11)	17	Yes	No
**2**	0	1	3	1	0	4			0	No	No
**164**	0	2	0	0	0	4	3 (n = 40)	5 (n = 114)	154	Yes	No
**40**	0	2	1	1	0	4			0	No	No
**1**	1	0	1	1	0	4			0	No	No
**450**	0	2	1	0	0	5	4 (n = 16)	6 (n = 54)	70	Yes	Yes, likely pathogenic
**162**	0	1	3	0	0	5		6 (n = 2)	2	Yes	Yes, likely pathogenic

Table includes 4,085 variants with VUS classification, presented alongside all ACMG evidence groups. There are 20 groups in total, encompassing different combinations of evidence categories: PS (pathogenic strong), PM (pathogenic moderate), PP (pathogenic supporting), BP (benign supporting), and BS (benign strong). Each item of evidence is quantified by assigning point adaptations, with ACMG categories considered benign if the score is ≤ −4 (dark blue in the table), likely benign if the score is −3 to −1 (light blue), VUS with a score 0–5 (yellow), LPATH 6–9 (light red), and PATH if ≥ 10 (dark red). VUS variants are further divided into three groups: “Low” (0–1 pts, light yellow), “Mid” (2–3 pts, yellow), and “High” (4–5 pts, gold yellow).

For each item of evidence, quantification is calculated by assigning a point adaptation: −4 points for BS and BA, −1 point for Benign-Supporting, 0 points for Indeterminate, 1 point for PP, 2 points for PM, 4 points for PS, and 8 points for PVS evidence. ACMG categories are labeled as benign if the score is ≤−4 (dark blue), likely benign if the score is −3 to −1 (light blue), VUS if 0–5 (yellow), 6–9 as likely pathogenic (light red), and ≥10 as pathogenic (dark red). According to the calculated quantification, 24 VUS variants lean toward likely benign (quantification: −1), and 4061 variants have uncertain significance (ranging 0–`5) (Table 5).

Additionally, all VUS variants are categorized into three groups: Low (0–1 pts, light yellow), Mid (2–3 pts, yellow), and High (4–5 pts, gold yellow). For VUS variants *without existing computational* evidence (BP4 for benign or PP3 for pathogenic), an AlphaMissense prediction was added to observe its influence on subcategory change and ACMG classification. Out of 4085 VUS variants, 1141 lacked BP4/PP3 evidence. AlphaMissense prediction, based on the AlphaMissense score, was included as evidence (308 were predicted as benign and 695 as pathogenic by AlphaMissense). After incorporating BP4 or PP3 evidence, the VUS quantification changed for 934 variants. Notably, the quantification for 12 VUS variants changed to likely benign (score: −1) after adding the AlphaMissense prediction, but the ACMG classification remained the same. For 56 variants, the quantification changed (score: 6) after including the AlphaMissense prediction as PP3 evidence, elevating the classification to likely pathogenic. For the remaining 866 variants, the quantification still indicated the VUS category, and their VUS subcategories changed depending on whether BP4 or PP3 evidence was assigned. The list of all reclassified VUS variants according to the quantification is listed in Supplementary Table S3.

The AlphaMissense scores for the 12 reclassified variants with benign predictions ranged from 0.0644 to 0.3204, while the pathogenic prediction scores for the 56 newly classified likely pathogenic variants were mostly above 0.9 (73%).

Since 75% of VUS variants showed concordance between the computational tools already used and AlphaMissense, we conducted a test to assess the impact of AlphaMissense as the sole computational evidence. We removed existing ACMG computational evidence from VUS variants and replaced it with AlphaMissense predictions. The replacement of AlphaMissense predictions, instead of existing computational evidence, resulted in a change in VUS quantification for 1709 variants. Notably, for 29 VUS variants, the quantification changed to likely benign (score: −1) after adding the AlphaMissense prediction, while the ACMG classification remained the same. For 63 variants, the quantification changed (score: 6) after including or replacing existing computational predictions with the AlphaMissense prediction or PP3 evidence, thereby elevating their classification to likely pathogenic (Table 6).

**TABLE 6:** Impact of AlphaMissense predictions on VUS variants when existing computational evidence is replaced by AlphaMissense predictions. VUS variants quantified through a points-based system using Bayesian framework (Tavtigian et al., 2020).

No. of variants	PS	PM	PP	BP	BS	Quantification after AlphaMissense replacement/addition	Quantification leading to reclassification
49	0	0	0	1	0	−1	No
1	0	0	1	2	0	−1	No
12	0	0	0	0	0	0	No
38	0	0	1	1	0	0	No
9	0	1	0	2	0	0	No
9	0	0	1	0	0	1	No
6	0	0	2	1	0	1	No
617	0	1	0	1	0	1	No
1	0	1	1	2	0	1	No
8	0	0	2	0	0	2	No
303	0	1	1	1	0	2	No
186	0	1	0	0	0	2	No
5	0	2	0	2	0	2	No
2	0	0	3	0	0	3	No
1,083	0	1	1	0	0	3	No
36	0	1	2	1	0	3	No
133	0	2	0	1	0	3	No
842	0	1	2	0	0	4	No
2	0	1	3	1	0	4	No
44	0	2	0	0	0	4	No
42	0	2	1	1	0	4	No
1	1	0	1	1	0	4	No
153	0	1	3	0	0	5	No
440	0	2	1	0	0	5	No
2	0	1	4	0	0	6	Yes, likely Pathogenic
61	0	2	2	0	0	6	Yes, likely Pathogenic

Table includes 4085 variants with VUS classification, presented alongside all ACMG evidence groups. There are 20 groups in total, encompassing different combinations of evidence categories: PS (pathogenic strong), PM (pathogenic moderate), PP (pathogenic supporting), BP (benign supporting), and BS (benign strong). Each item of evidence is quantified by assigning point adaptations, with ACMG categories considered benign if the score is ≤ −4 (dark blue in the table), likely benign if the score is −3 to −1 (light blue), VUS with a score 0–5 (yellow), LPATH 6–9 (light red), and PATH if ≥ 10 (dark red). VUS variants are further divided into three groups: “Low” (0–1 pts, light yellow), “Mid” (2–3 pts, yellow), and “High” (4–5 pts, gold yellow).

**FIGURE 4:**
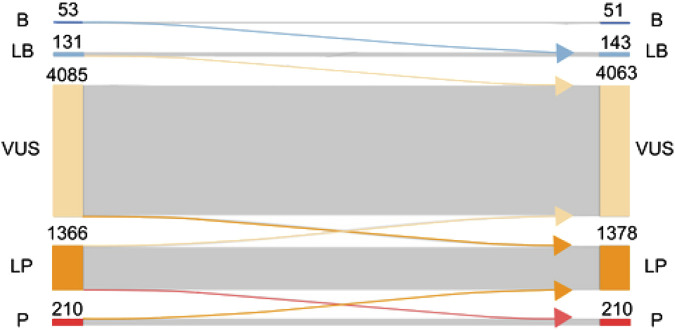
Effect of replacing existing computational evidence with AlphaMissense predictions on the final classification. ACMG classification of variants (B, benign; LB, likely benign; VUS, variant of unknown significance; LP, likely pathogenic; P, pathogenic) is presented on the left side before AlphaMissense predictions are added to the classification. Once AlphaMissense predictions are incorporated into the classification, the number of benign, likely benign, VUS, likely pathogenic, and pathogenic variants is shown. Arrows going from left to right show the number of variants changing classification after the incorporation of AlphaMissense predictions.

When computational evidence for all variants was removed and replaced with AlphaMissense predictions, 194 variants were benign/likely benign (51 were benign and 143 were likely benign), 1588 variants were pathogenic/likely pathogenic (1,378 were likely pathogenic and 210 were pathogenic), and 4063 variants were classified as VUS (Figure 4). In total, 142 variants changed classification, which is 2.4% of all variants. More specifically, two variants changed from benign to likely benign, one likely benign variant became VUS, 11 VUS variants were reclassified as likely benign, 63 VUS variants as likely pathogenic, seven likely pathogenic variants as pathogenic, 51 likely pathogenic variants were reclassified as VUS, and seven pathogenic variants as likely pathogenic.

## Discussion

The accurate classification of genetic variants is crucial for the proper diagnosis, prognosis, and therapy of genetic diseases. Although many genetic variants are associated with known clinical significance and treatment, more than a third of all variants are categorized as variants of uncertain significance (VUS), presenting a challenge to clinicians, geneticists, and patients due to the ambiguity surrounding their impact on health and disease. ACMG/AMP guidelines categorize variants into benign, likely benign, VUS, likely pathogenic, and pathogenic using several different classes of evidence that include population data, functional data, computational *in silico* predictions, segregation data, *de novo* data, and allelic and other data. AlphaMissense is a promising new AI-based pathogenicity prediction tool developed by Google’s DeepMind which combines AI, structural information, and evolutionary conservation to predict the functional impact of missense variants. With the power of AI to integrate multiple levels of evidence, it may be plausible that tools such as AlphaMissense may provide better discriminatory power in elucidating the classification of VUS variants as the most challenging class of variants. Thus, the primary objective of this study was to evaluate the predictive accuracy and reliability of AlphaMissense in characterizing missense variants in a curated set of 59 genes associated with neurological, musculoskeletal, and/or neuromuscular disorders through comparative analysis between AlphaMissense predictions and the adapted ACMG classification provided by Genomenon’s Mastermind platform.

The analysis of 5845 missense variants in 59 genes revealed notable disparities between pathogenicity predictions by AlphaMissense and predictions based on the ACMG guidelines in Mastermind. The overall assessment of sensitivity and specificity when pathogenic versus benign calls are compared indicates that AlphaMissense is successful in identifying variants classified as P/LP by ACMG classification in Mastermind and operates well in identifying B/LB ACMG-classified variants. The high positive predictive value suggests that variants predicted as *pathogenic* by AlphaMissense are indeed *pathogenic/likely pathogenic* according to ACMG classification; however, the overall NPV is lower, implying that variants predicted as *benign* by AlphaMissense might have a higher chance of being misclassified when compared to ACMG. When analyzed within the context of VUS, AlphaMissense showed a low specificity, indicating that it was ineffective in identifying *VUS* variants as classified by ACMG. Similarly, poor PPV revealed that variants predicted as *pathogenic* have a slim chance of being truly *pathogenic/likely pathogenic.* A moderately high negative predictive value indicated that AlphaMissense was effective in predicting ambiguous variants.

The results for individual genes vary; AlphaMissense showed overall good performance in predicting variants of the SCN4A, NF1, DOK7, and FKRP genes. GRIN2A and RYR1, however, exhibited lower specificity and NPV, emphasizing the importance of gene-specific assessments. The negative predictive value is consistently less than 50% in all individually reviewed genes, which underscores the overall result and indicates the challenges in using AlphaMissense’s data regarding variants predicted as benign.

VUS variants classified by assigning a substantial amount of pathogenic and benign evidence were often predicted as benign by AlphaMissense, indicating potential overcalls. VUS variants that were classified as benign by AlphaMissense were in most cases what is considered a “VUS plus” or a “warm” VUS variant, which only must fulfill one additional pathogenic criterion to be promoted to a likely pathogenic classification but lacked one at the time of the first classification attempt.

Variants predicted ambiguous by AlphaMissense were mostly concordant with ACMG classification, but a notable segment was classified as pathogenic/likely pathogenic by Mastermind, based on the fact that almost no variant met benign criteria. Variants with conflicting classifications and predictions, pathogenic/benign by Mastermind and benign/pathogenic by AlphaMissense , stress the intricacies of variant interpretation and the need for a comprehensive evaluation of conflicting evidence through extensive research and use of helping tools instead of solely relying on predictions.

The re-classification of VUS in genetic testing is critical to improving our understanding of genetic variations and their potential impact on health. As depicted in Figure 3, among the 1019 VUSs categorized by ACMG without assigned computational evidence, AlphaMissense predicted 314 as benign and 705 as pathogenic. By considering the AlphaMissense benign predictions as BP4 evidence, which signifies computational inertness, four (1.3%) were reclassified as likely benign during the interpretation of the 314 variants. Conversely, applying PP3 evidence—denoting computational prediction of damage—to the 705 VUS variants based on AlphaMissense pathogenic predictions resulted in the promotion of 52 variants (7.4%) to likely pathogenic. Other studies indicated that approximately 10%–15% of re-evaluated VUS could be elevated to the category of likely pathogenic/pathogenic, while the remaining cases may be reclassified as likely benign/benign (Burke et al., 2022). Our analysis using AlphaMissense predictions indicates a lower percentage (7.4%) of variants being promoted to likely pathogenic compared to the general estimate (10%–15%). Thus, the use of computational evidence (AlphaMissense predictions) plays a significant role in both reclassifications, with computational inertness leading to some benign variants being reclassified as likely benign and computational prediction of damage promoting some VUS variants to likely pathogenic.

The correlation between AlphaMissense scores and ACMG classification was evident, with quantified ACMG pathogenic and benign variants aligning well with high and low AlphaMissense scores, respectively. The quantification process assigned scores based on the type and strength of evidence, establishing a continuum from benign to pathogenic. This allowed for a nuanced characterization of all variants—particularly of VUS variants. Notably, 24 VUS variants were identified as likely benign if the quantification system was used, while the majority of 4061 variants retained an uncertain significance, with a 0–5 score range.

The stratification of VUSs based on the quantification scores (low, mid, and high) provided additional insights into the distribution of variants across different quantification levels and the ability to better track the possible effects of AlphaMissense predictions. When we explored the impact of AlphaMissense predictions on VUS variants that lacked existing computational evidence (BP4 for benign or PP3 for pathogenic), we incorporated AlphaMissense predictions into the analysis and found that of the 4085 VUS variants, 1141 lacked BP4/PP3 evidence. Furthermore, after integrating AlphaMissense predictions, 308 variants were predicted as benign and 695 as pathogenic. Subsequent analysis revealed notable changes in the quantification of VUS variants, where 878 variants changed quantification within the VUS framework. Remarkably, for 12 VUS variants, the quantification shifted to likely benign after incorporating AlphaMissense predictions, although the ACMG classification remained the same. This suggests that while the quantification changed, the overall pathogenicity assessment remained consistent. On the other hand, for 56 variants, the quantification increased to a score of 6, leading to an elevation in ACMG classification to likely pathogenic. This highlights the substantial impact of AlphaMissense predictions on reclassifying certain variants, emphasizing their potential contribution to refining pathogenicity assessments. This underscores the complex interplay between computational evidence, AlphaMissense predictions, and existing ACMG criteria in determining the significance of genetic variants.

An intriguing aspect of the study is the examination of scenarios where AlphaMissense predictions were the sole computational evidence, replacing existing ACMG computational evidence. The results showed that this replacement led to changes in VUS quantification for 1079 variants, emphasizing the significance of AlphaMissense as an independent computational tool in the variant interpretation of VUS.

The use of AlphaMissense as a predictive tool did not result in a significant shift in current ACMG classification. Of the total number of VUS variants (n = 4,085), a small number (n = 63) were reclassified into likely pathogenic after incorporating AlphaMissense as computational evidence. It is important to note that 51 variants initially classified as likely pathogenic moved into the VUS category. Therefore, the overall number of variants that changed classification with the help of AlphaMissense was 114, leading to the conclusion that the classifications essentially align and there is no significant impact. It is also crucial to emphasize that AlphaMissense is a stronger predictive tool, primarily because it incorporates functional and population data. After the analysis and incorporation of AlphaMissense as a predictive tool, the recommendation is to classify variants according to ACMG standards. However, VUS variants should undergo a completely new classification approach. The central database should be AlphaMissense, which should continuously improve its function with new evidence and data. One noticeable effect is that AlphaMissense influenced the quantification of VUS variants, causing their transition from low to mid and high VUS levels and *vice versa*.

Other studies have shown that AlphaMissense is highly effective in predicting pathogenicity for both somatic variants, particularly in hematological malignancy diagnostics. A study with 686 patients across 111 genes demonstrated its accuracy, achieving an impressive AUC of 0.95 (Chabane et al., 2024). Compared to 38 other predictors using multiplexed assays of variant effect (MAVE), AlphaMissense showed strong correlation with functional impact, especially for specific genes (Ljungdahl et al., 2023). The analysis against Rhapsody and its performance based on AlphaFold structures further establishes its prominence. Utilizing advanced structural information, AlphaMissense generates higher pathogenic probabilities, validated by identifying annotated pathogenic variants in the ClinVar database (Wang et al., 2023; Staklinski et al., 2023; Ahmad et al., 2024; McDonald et al., 2024).

Current computational models for predicting the pathogenicity of missense variants have been valuable for interpreting genetic variants. However, these models face several challenges and limitations, including the size of the dataset, bias in training data, and difficulties with rare variants. REVEL and PolyPhen rely on publicly available variant databases (e.g., ClinVar) and large population cohorts (e.g., gnomAD) to train their algorithms. However, many of these databases are incomplete or biased toward common variants. Many of the available pathogenic variant databases are biased toward well-studied diseases and populations (e.g., European ancestry), which leads to skewed predictions for other populations. The number of known pathogenic variants is relatively small, which leads to challenges in training highly accurate models, especially for rare and ultra-rare variants. Consequently, these models tend to perform well with common variants but struggle to generalize to unseen or rare variants. Thus, these models often struggle with rare variants, especially variants that have never been seen before in training datasets because these models rely on known pathogenic or benign labels to make predictions, and rare variants are less likely to have established labels.

AlphaMissense could hold potential to influence clinical decision-making in ways that go beyond ACMG classification guidelines. One option is that the new ACMG guidelines offer computational algorithms with more value than just supporting evidence, allowing for their greater impact on variant classification, particularly VUS. If AlphaMissense predictions were to become more reliable, AlphaMissense could integrate with broader medical guidelines and clinical workflows in cancer oncology, pharmacogenomics, hereditary cancer screening, and newborn screening. However, it is notable that despite being deemed the current best-in-class predictor, only modest improvements over other algorithms were reported. The studies also indicate that combining predictions from multiple tools, including SIFT4G and REVEL, did not significantly enhance performance compared to using AlphaMissense alone. This suggests that AlphaMissense offers robust predictive capabilities on its own, reducing the necessity for complex ensemble approaches in certain contexts (Nadya et al., 2023). Our results also indicate that the classifications essentially align and there is no significant impact on variant classifications when different prediction tools are used.

## Conclusion

AlphaMissense could hold potential to influence clinical decision-making in ways that go beyond ACMG classification guidelines. One option is that the new ACMG guidelines offer computational algorithms more value than just supporting evidence, allowing for their greater impact on variant classification, particularly VUS. If AlphaMissene predictions become more reliable in the future, AlphaMissense could integrate with broader medical guidelines and clinical workflows in cancer oncology, pharmacogenomics, hereditary cancer screening, and newborn screening. Overall, the multifaceted performance of AlphaMissense suggests its significant potential in guiding diagnostic and therapeutic strategies, contributing valuable insights to the field of precision medicine.

## Data Availability

The datasets presented in this study can be found in online repositories. The names of the repository/repositories and accession number(s) can be found in the article/Supplementary Material.
